# Ethical Considerations for Discrete Choice Experiments with Caregivers

**DOI:** 10.1177/15562646221112339

**Published:** 2022-07-18

**Authors:** Judy Illes, Ashley Lawson, Patrick J. McDonald

**Affiliations:** 1 Division of Neurology, Department of Medicine, Neuroethics Canada, University of British Columbia, Vancouver, Canada; 2 Section of Neurosurgery, Department of Surgery, Max Rady College of Medicine, University of Manitoba, Winnipeg, Canada

**Keywords:** research ethics, neuroethics, vulnerable populations, epilepsy, discrete choice experiment

## Abstract

We discuss research ethics challenges experienced while running a discrete choice experiment administered to caregivers of children with treatment resistant pediatric epilepsy. We highlight ethical considerations around the study design of the discrete choice experimental paradigm that pertain to vulnerability of and caregiving burden on the population, imbalance of benefit-to-load of participation, and limitations of cultural meaningfulness and generalizability.

Discrete choice experiments (DCE) have been used since 1983 to identify preference-sensitive variables where multiple, equally valid options exist (Louviere & Woodworth, 1983). DCEs force choices between hypothetical conditions, described by key features known as attributes. They have been applied in a wide range of disciplines – marketing, economics, and ecology– and more recently, have gained prominence in health-related research (Elwyn et al., 2009; Organisation for Economic Co-operation and Development, 2018; Ryan, 2004). From the latter, results have yielded new knowledge about valuing patient experiences, investigating trade-offs in health outcomes and patient experiences, and developing priority-setting frameworks with a direct impact on treatment (Soekhai et al., 2019). In these circumstances, the best option depends on patients, together with their clinicians, choosing a treatment that aligns best with their preferences. As described by Apakantu et al. (2021), these preferences can be influenced by outcomes (i.e., potential benefits and harms), as well as processes (e.g., the way treatment is delivered), and the context (i.e., structures) in which treatment is delivered (e.g. the health care team). For example, Tamber and Naftel (2020) conducted a study that used a modified DCE to elicit treatment preferences from parents of children with hydrocephalus. By asking parents if they would accept various hypothetical treatments – each with a perceived risk attribute paired with a perceived benefit attribute – the authors found that the most important drivers of treatment choice were surgical risk, minimizing repeat operations, and maximizing cognitive outcomes (Tamber & Naftel, 2020).

Even the best designed medical research with human participants will fail, however, if not well-suited to its target population, and this includes the full range of ethical considerations from consent to confidentiality. While there exist ethics guidelines to assist researchers in applying ethical principles (CIOMS, 2016), it is the duty of researchers to be cognizant of and manage ethical considerations both in advance of, and as they arise during the course of research. In a study of caregivers of children with drug resistant epilepsy (DRE), our goal was to collect information from both physicians and caregivers on the attributes they value when deciding on an intervention that involves a neurotechnological approach such as vagal nerve stimulation (VNS), responsive neurostimulation (RNS) and deep brain stimulation (DBS), among others. Such interventions include invasive, high maintenance, albeit reversible implantation in the brain (DBS) or nerves (VNS), or non-reversible, low maintenance approaches involving destruction of epileptogenic tissue.

We were successful in obtaining a sufficient number of responses with the physician DCE based on the preparatory qualitative study (McDonald et al., 2021), but unsuccessful in obtaining enough responses in a caregiver DCE even though the attributes were derived, and DCEs designed, using the same methodology. The contributions to this failure, and focus of this brief report, involve not only a possible methodological mismatch with the target population, but considerations that fall squarely within the realm of research ethics.

## Methodological Approach

The first step to the caregiver DCE study was to determine which attributes were most important to the target population. DCEs are underpinned by random utility theory - Lancaster’s theory - of demand (McFadden, 1973; Sargan, 1972). The theory provides a framework for preference elicitation methodologies by distilling decision-making into its component parts, i.e., decision-making about goods and services can be decomposed into combinations of their attributes and levels, each with different values. Guidelines on the development of DCEs recommend that relevant attributes and levels should be identified by qualitative research (Lancsar & Louviere, 2008).

In this initial qualitative study, caregivers with affected children were recruited to semi-structured focus groups or interviews at one of four major epilepsy centers in Eastern and Western Canada and the USA (n = 22) (Hrincu et al., 2021). Consent was obtained in advance of participation in written (digital) form through email, and affirmed at the start of each interview. Discussions were transcribed and qualitative analytic methods applied to examine values and priorities (e.g., risks, benefits, adherence, invasiveness, reversibility) of caregivers pertaining to novel technologies to treat DRE. An inductive approach was used to identify major themes: 1) *features of the intervention*: risks and benefits, with an emphasis on an aversion to perceived invasiveness; 2) *decision drivers*: trust in the clinical team, treatment costs; and, 3) *resources*: quality of available information about neurotechnological options. Overall, caregivers’ definition of treatment success is more expansive than the single variable of seizure freedom favored by clinicians.

The DCE based on these results was designed by an interdisciplinary team of health economists, ethicists, clinicians and health service researchers. In developing the full set of 6-8 attributes for the experiment, the core question was: “*Does this drive decision-making*?” Selecting attributes is a balance between including the most important features while limiting the number of attributes so that the complexity of the tasks for the respondent is manageable. The risk, otherwise, is non-attendance, inconsistent responses across choice tasks, and increased drop-out rates (Obadha et al., 2019).

The experts and researchers screened all generated attributes and levels using multiple criteria such as decision context and plausibility. As a decision to choose the option of “no intervention” was plausible, a no intervention opt-out was carefully defined. Multiple versions of the DCE were developed, vetted, and refined by the research team as well as select caregiver stakeholders who participated in the focus groups and consented to recontact. Consent for the DCE was obtained via a form embedded in the first page of the survey alongside survey details and information about the study. In testing, time to completion was an average of 25 min. The final version was translated from English into both French and Spanish for maximum inclusivity of prospective respondents residing in both Canada and the USA. Minimum N to power the analysis was 70 from a possible pool of 150,000.

Dissemination of the DCE to caregivers was conducted via recruitment posters in epilepsy clinics at the four sites involved with the caregiver focus groups featuring scannable QR codes, as well as via a dissemination partner at the Brain Recovery Project who shared the survey links to online patient support networks and social media platforms. An invitation to caregivers to complete the DCE was posted on the Brain Recovery blog and Facebook page and reposted a month later as a follow-up, shared with pediatric epilepsy surgery support groups, and the rare epilepsies network representing 70 patient advocacy groups. The project was approved by the University of British Columbia Behavioral Research Ethics Board (BREB) # H18-02783.

## Outcomes

Nine responders completed the English-language DCE choice sets, including the demographics questions; 10 completed only the DCE choice sets; 15 went past the introduction and consent pages but failed to proceed into the choice sets; 5 began the choice sets and but terminated before completion. There were no responses to either the French or Spanish versions.

## Observations and Discussions

While the simplicity of the DCE exercise and the familiarity of making choices in real-life situations has been cited as important strengths of the approach (Ryan & Farrar, 2000), the response numbers were insufficient to power a meaningful analysis. Upon closing the study, we received feedback from collaborators engaged to assist with dissemination of the DCE that the real-life burden of caring for children with DRE, and the out-of-context nature of the experiment that included the hypothetical treatment options, were major impediments to participation. The unpredictable nature of seizures constitutes a major stressor for caregivers. The additional pressures on and lack of social supports during the recent pandemic may have exacerbated this condition (Boreale, 2020).

While we took into account strategies and recommendations of past studies on the methodology of DCE that have addressed ways to reduce cognitive load and low response rates (Bryan & Dolan, 2004; Coast et al., 2012), the choice task still involves a considerable cognitive challenge as respondents are required to process large amounts of information contained in the scenarios and consider trade-offs between all of the attributes. Decreasing the number of attributes from 6-8 to 2-4 might have increased response rate, but the significance of the experiment, and the generalizability of the results, would decrease proportionally (Watson et al., 2017). Further, within the healthcare setting, choices may be less familiar to respondents than, for example, marketing choices, making each choice more burdensome or complex such that individuals more quickly reach a threshold beyond which they are unwilling to participate, and thus fail to complete the DCE (Viney et al., 2005). Compounding the issue, cognitive load has already been heightened by the ongoing COVID-19 pandemic and the implications for caregivers of young children navigating care and safety of their school-aged children (Masi et al., 2021; Vaillancourt et al., 2021). We did not offer any incentive to participate. The direct effect or interaction of these choices with the current public health crisis is unknown.

We note independently that the requirement for only 6-8 attributes all ultimately related to biomedical aspects of candidate neurotechnologies, and excluded variables involving diverse cultural views about interventions on the brain. For participants for whom wholly biomedical explanations of disease is not sufficient, or for those living in rural and remote geographic regions with challenging access to advances to new technologies (Harding & Illes, 2021), the relevance of the DCE was possibly too low. Indeed, should the study actually have recruited sufficient responses to power the analysis, adoption of results would have excluded their views at best; at worst, they would only serve to further stigmatize and marginalize them, and increase a gap in already well documented health disparities.

## Best Practices

The suitability of DCEs to certain vulnerable populations, however vulnerability is defined, is a matter not only of economics and science, but of research ethics. Respect for persons is violated if burden – i.e., risk – exceeds benefit. Context matters. Inclusivity is at stake given the technical limitations and cognitive load elements of the approach. Meeting the goal of justice is a challenge if implementation falls short of meaningfulness. More discussion surrounding appropriate methodology and patient engagement during study planning and design that draws upon CIOMS 2016 Guideline 7, for example, as well as institutional review is needed to ensure that the highly validated, quantitative approach of the DCE method also meets the highest and broadest standards of ethics in research.

## Research Agenda

The discussion above highlights an under estimation of the cognitive burden and vulnerability of caregiving populations that may limit, or at least challenge, the appropriateness of DCE methodology. A means to assess the vulnerability of caregiving populations prior to the onset of research outset would lead to special design features and protections if necessary (CIOMS, 2016). The discussion also brings into question the cultural relevance of the approach, and the ethically suitable interpretations of data even when the responses are sufficient to power analysis. Further studies that directly investigate these phenomena are needed to provide evidence-based modifications to the method to yield interpretable results while maintaining non-maleficence. Further research on other populations of caregivers, such as caregivers of older adults or peers, would also enhance the understanding and ethical application of the DCE methodology.

## Educational Implications

The responsibility of investigators to choose and fully disclose not only the proper scientific method to answer a research question, but also one that is ethically best suited to a population of interest was discussed in *Ethical Reproducibility* by Anderson et al. (2013). By reporting the challenges encountered over the course of the research here, we model ethical reproducibility that speaks to the importance of communicating not only scientific methods of a research study, but its ethical features. Our experience with a DCE of caregivers with children with DRE empirically reaffirms the importance of the continued movement from a science-now-ethics-later divide, to proactive science-ethics integration that considers, upfront, the context and lived experience of participants.
